# *Melicope ptelefolia* leaf extracts exhibit antioxidant activity and exert anti-proliferative effect with apoptosis induction on four different cancer cell lines

**DOI:** 10.1186/s12906-017-1761-9

**Published:** 2017-05-05

**Authors:** Mohammad Faujul Kabir, Johari Mohd Ali, Mitra Abolmaesoomi, Onn Haji Hashim

**Affiliations:** Department of Molecular Medicine, Faculty of Medicine, University of Malaya, 50603 Kuala Lumpur, WP Malaysia

**Keywords:** *Melicope ptelefolia*, Antioxidant activity, Cellular antioxidant assay, Anticancer, Apoptosis, Cell cycle, HCT116, HCC1937, HepG2, MDA-MB-231

## Abstract

**Background:**

*Melicope ptelefolia* is a well-known herb in a number of Asian countries. It is often used as vegetable salad and traditional medicine to address various ailments. However, not many studies have been currently done to evaluate the medicinal benefits of *M. ptelefolia* (MP). The present study reports antioxidant, anti-proliferative, and apoptosis induction activities of MP leaf extracts.

**Method:**

Young MP leaves were dried, powdered and extracted sequentially using hexane (HX), ethyl acetate (EA), methanol (MeOH) and water (W). Antioxidant activity was evaluated using ferric reducing antioxidant power (FRAP), 2,2′-azinobis-(3-ethylbenzothiazoline-6-sulfonic acid) (ABTS) and 1,1-Diphenyl-2-picryl-hydrazyl (DPPH) radicals scavenging and cellular antioxidant activity (CAA) assays. Anti-proliferative activity was evaluated through cell viability assay, using the following four human cancer cell lines: breast (HCC1937, MDA-MB-231), colorectal (HCT116) and liver (HepG2). The anti-proliferative activity was further confirmed through cell cycle and apoptosis assays, including annexin-V/7-aminoactinomycin D staining and measurements of caspase enzymes activation and inhibition.

**Result:**

Overall, MP-HX extract exhibited the highest antioxidant potential, with IC_50_ values of 267.73 ± 5.58 and 327.40 ± 3.80 μg/mL for ABTS and DPPH radical-scavenging assays, respectively. MP-HX demonstrated the highest CAA activity in Hs27 cells, with EC_50_ of 11.30 ± 0.68 μg/mL, while MP-EA showed EC_50_ value of 37.32 ± 0.68 μg/mL. MP-HX and MP-EA showed promising anti-proliferative activity towards the four cancer cell lines, with IC_50_ values that were mostly below 100 μg/mL. MP-HX showed the most notable anti-proliferative activity against MDA-MB-231 (IC_50_ = 57.81 ± 3.49 μg/mL) and HCT116 (IC_50_ = 58.04 ± 0.96 μg/mL) while MP-EA showed strongest anti-proliferative activity in HCT116 (IC_50_ = 64.69 ± 0.72 μg/mL). The anticancer potential of MP-HX and MP-EA were also demonstrated by their ability to induce caspase-dependent apoptotic cell death in all of the cancer cell lines tested. Cell cycle analysis suggested that both the MP-HX and MP-EA extracts were able to disrupt the cell cycle in most of the cancer cell lines.

**Conclusions:**

MP-HX and MP-EA extracts demonstrated notable antioxidant, anti-proliferative, apoptosis induction and cancer cell cycle inhibition activities. These findings reflect the promising potentials of MP to be a source of novel phytochemical(s) with health promoting benefits that are also valuable for nutraceutical industry and cancer therapy.

**Electronic supplementary material:**

The online version of this article (doi:10.1186/s12906-017-1761-9) contains supplementary material, which is available to authorized users.

## Background

The plant kingdom is an essential source of novel phytochemicals for drug discovery. About 25% of the drugs prescribed worldwide originated from plants [1]. Despite such fact, only a small percentage of plant species have been scientifically studied to date, for isolation of phytochemicals of medical importance [1]. Plant species demonstrating antioxidant and anticancer activities are important in nutraceutical and pharmaceutical industries, for they are considered as valuable sources of dietary phytochemicals with medicinal and health-promoting properties. Dietary intake of herbs that are rich with antioxidants may counteract oxidative stress, and this may help reduce the risk of acquiring chronic diseases such as cancer [2].

The present study was carried out to investigate the potential medicinal benefits of *Melicope ptelefolia* (MP), namely its antioxidant and anticancer activities. This study may eventually lead to the isolation of novel phytochemicals from MP that are of importance for nutraceutical and cancer therapeutics industries. MP belongs to the family of Rutaceae and it is a widely renowned herb in Asian countries. It is known as ‘tenggek burung’, ‘sampang Uam’ and ‘Uam, Sam Ngam’ in Malaysia, Indonesia and Thailand, respectively [3]. Fresh MP leaves have a slight crunchy texture and a pleasant hint of refreshing lemon-lime aroma that is mildly pungent, hence its popularity being used as a vegetable salad. Traditionally, MP has been used to address various ailments such as fever, rheumatism, stomach ache, wounds, and itches [4]. However, the full potential of its medicinal benefits has not yet been exhaustively investigated. MP leaves and roots have been reported to show anti-nociceptive and anti-inflammatory activities [5, 6]. Seven compounds have been identified from the Malaysian species of MP leaves [7], whereby 2,4,6-trihydroxy-3-geranylacetophenone (tHGA) was one of the compounds reported to show anti-inflammatory activity [8]. Melicolones A and B, isolated from MP leaves were reported to inhibit glucose induced oxidative damage in HUVEC cells [9].

In the present study, young leaves of MP were dried and sequentially extracted using four solvents of varying polarities, namely hexane, ethyl acetate, methanol and water. To the best of our knowledge, this extraction method has never been reported in the study of MP. Characterization of antioxidant activity of the extracts was performed based on chemical antioxidant activity methods and cell based antioxidant assay. The anti-proliferative and apoptosis induction activities were investigated using HCT116, HCC1937, MDA-MB231 and HepG2 cancer cell lines.

## Methods

### Reagents, solvents and chemicals

The reagents and chemicals used in this study were of analytical grade and mainly obtained from Fisher Scientific, Sigma-Aldrich and Merck-Millipore. Tissue culture media were purchased from Nacalai Tesque.

### Sample preparation

Fresh and healthy MP young leaves were purchased from the local wet market. A voucher specimen was deposited at the University of Malaya (UM) herbarium (Rimba Ilmu, Institute of Biological Sciences, UM) and the sample identity was also authenticated by the herbarium’s botanist, Dr. Sugumaran Manickam. The leaves were washed with distilled water and air dried until no weight reduction was observed. The dried leaves were powdered using a table blender and stored at −20 °C until needed for the extraction. Organic raspberry, blueberry and blackberry were purchased from a local supermarket, washed with distilled water and dried in a 40 °C oven until no weight reduction was observed. They were powdered using a table blender and stored at −20 °C until needed for the extraction.

### Extracts preparation

Powdered dried MP leaves were extracted sequentially, using solvents of varying polarity in following order: hexane > ethyl acetate > methanol > water. Fifty grams of the powdered leaves was mixed with 500 mL of hexane and the extraction was carried out by incubating the mixture in an incubator shaker at 37 °C for 6 h. The supernatant was obtained by centrifugation at 1500 rpm for 10 min, followed by filtration using a Whatman filter paper (No. 4). The extraction using hexane was repeated twice, and the residues were dried. The extraction was then continued using the remaining three solvents following the method as indicated above. The resulting 1500 mL of solvent collected for each extraction was evaporated using a rotary evaporator. The water extract was evaporated using a freeze dryer. The same extraction procedure was performed for the berries fruit. The dried extracts were dissolved in 10% DMSO at 2 mg/mL and stored at −20 °C.

### Cell lines and tissue culture protocol

The following American type culture collection (ATCC) human cancer/normal cell lines were used: HCT116, HCC1937, HepG2, MDA-MB-231, CCD841, Hs27. The cell lines were maintained according to the ATCC guidelines. Growth medium was supplemented with 10% foetal bovine serum (FBS) and 1% penicillin-streptomycin. TrypLE™ Express enzyme (Gibco) was used for cells detachment. Incubation of culture was at 37 °C, in a humidified incubator containing 5% CO_2._

### Antioxidant potential assays

#### Total Phenolic Content

Total polyphenol content (TPC) was determined using Folin-Ciocalteu (FC) reagent [10]. Twenty μL of the plant extracts were each mixed with 100 μL of 10% FC reagent in a 96-well plate and incubated for 30 min. Seventy μL of Na_2_CO_3_ (1 M) was added to the mixture and incubated for 2 h at room temperature. Absorbance was then measured at 735 nm. A standard curve was constructed using gallic acid (0–0.250 mg/mL). The TPC value was expressed as mg gallic acid equivalents per gram of dried extract.

#### Total flavonoid content

Total flavonoid content (TFC) was determined according to Chang et al. [11] with slight modifications. Five μL each of aluminium trichloride (10% *w*/*v*) and potassium acetate (1 M) were mixed with 25 μL of the plant extract. Seventy-five μL of ethanol (95% *v*/v) and 140 μL of distilled water were added to the mixture. The mixture was incubated for 30 min at room temperature followed by absorbance reading at 415 nm. Quercetin was used as a standard and TFC values were expressed as mg quercetin equivalent per gram of dried extract.

#### Ferric reducing antioxidant power (FRAP)

FRAP assay [12] was carried out using freshly prepared FRAP reagent by mixing 10 mM TPTZ (2,4,6-tripyridyl-s-triazine) in 40 mM HCl, acetate buffer (300 mM, pH 3.6) and FeCl_3_.6H_2_O (20 mM) in 1:10:1 ratio (*v*/v/v). Ten μL of the plant extract was mixed with 300 μL FRAP reagent and incubated for 4 h at room temperature. The absorbance of ferrous-TPTZ complex was measured at 593 nm. A standard curve using FeSO_4_ solution (0 to 3.5 mM) was plotted. Antioxidant power of the extracts was expressed as mmol Fe^2+^ per gram of dried extract. Gallic acid, ascorbic acid and catechin were used as positive controls.

#### ABTS^●+^ radical-scavenging activity

The ABTS^●+^ (2,2′-azinobis-3-ethylbenzothiazoline-6-sulphonic acid) scavenging activity was performed according to Re et al. [13]. ABTS^●+^ cation was produced by mixing 7 mM ABTS solution with 2.45 mM potassium persulfate and allowing them to react in the dark for 16 h at room temperature. The absorbance of solution was measured at 734 nm and adjusted to ~0.7 using absolute ethanol. Ten μL of the plant extract at different concentrations (up to 2000 μg/mL) was mixed with 90 μL of ABTS^●+^ solution and the absorbance at 734 nm was taken after 30 min. Trolox, ascorbic acid, catechin and quercetin were used as positive controls. ABTS^●+^ scavenging activity was calculated as follows: ABTS^●+^ radical-scavenging activity = [(A_control_-A_sample_)/A_control_] X 100%, where A_control_ and A_sample_ are the absorbances in the absence and presence of extracts, respectively. The IC_50_ value is the concentration at which 50% of the ABTS^●+^ is scavenged. IC_50_ concentration of the extracts was determined using GraphPad Prism version 5.0 (GraphPad Software, Inc., USA).

#### DPPH• radical-scavenging activity

The DPPH• **(**2,2-diphenyl-1-picryl hydrazyl) radical-scavenging activity was evaluated according to Sharma and Bhat [14] with slight modifications. One hundred μM DPPH• solution was prepared in methanol and incubated at room temperature in the dark for 30 min. Twenty μL of plant extracts, at different concentrations up to 2000 μg/mL, was mixed with 150 μL of 100 μM DPPH• solution. The absorbance of the reaction mixture was measured at 517 nm after 30 min of incubation at room temperature. Ascorbic acid, quercetin, catechin and trolox were used as positive controls. The scavenging activity of the extracts was calculated as follows: DPPH• radical-scavenging activity = [(A_control_-A_sample_)/A_control_] X 100%, where A_control_ and A_sample_ are the absorbances in the absence and presence of extracts, respectively. The IC_50_ value is the concentration of the extracts that inhibited 50% of DPPH•, and this value was determined using GraphPad Prism version 5.0.

### Cellular antioxidant activity assay

The CAA assay was carried out according to Wolfe and Liu [15] with minor modifications. Briefly, Hs27 cells were seeded in 100 μL DMEM media at a density of 5 × 10^4^ cells/well in a black 96-well plate. The cells were then incubated for 24 h, after which the growth medium was removed and the wells were washed with cold PBS. The wells were applied with 100 μL medium containing MP extracts (up to 1000 μg/mL) and 25 μM 2′,7′-dichlorofluorescin diacetate (DCFH-DA) and incubated for 1 h. The cells were then washed with 100 μL of cold PBS. One hundred μL of 600 μM 2,2′-azobis (2-amidinopropane) dihydrochloride (ABAP) in Hank’s Balanced Salt Solution (HBSS) was applied to the cells and the fluorescence reading was recorded using Tecan Multimode reader & HydroFlex microplate washer (Infinite® M1000 Pro, Tecan Trading AG, Switzerland). Emission at 538 nm was measured with excitation at 485 nm every 5 min for 90 min. The assay included a triplicate control and blank. The control wells consisted of treated cells with DCFH-DA and ABAP while the blank contained cells treated with the dye and HBSS only. After blank subtraction from the fluorescence readings, the area under the curve of fluorescence versus time was integrated to calculate the CAA value according to the following formula: CAA unit = 100-(∫SA ⁄∫CA) × 100 [15]. The median effective dose (EC_50_) for the pure phytochemical compounds (positive controls) and MP extracts were determined from the median effect plot of log (*f*_*a*_ */f*_*u*_) versus log (dose), where *f*_*a*_ is the fraction affected and *f*_*u*_ is the fraction unaffected by the treatment, respectively [15]. The EC_50_ value of extracts was obtained when the ratio of ƒ_a_/ƒ_u_ equals to 1.

### Cell viability assay

The anti-proliferative activity of the extracts against cancer and normal cell lines was evaluated using Promega CellTiter 96® AQueous One Solution Cell Proliferation Assay (MTS) kit, according to the manufacturer’s instructions. The cells were seeded at 37 °C at a density of (~1 × 10^4^) cells/well/100 μL media in 96-well plate. In the preliminary screening, cells were treated with the extracts at 250 μg/mL. After 48 h, 20 μL of MTS reagent was added and the mixture was incubated for another 4 h, after which absorbance reading at 490 nm was taken. Cell viability was determined as follows: Viability (%) = (Absorbance of sample/Absorbance of control) X 100%. The plant extract that reduced cancer cells viability significantly (< 50%) were taken as positive for anti-proliferative activity, and was further investigated. The cancer cells were treated with the positive MP extracts for 48 h at various concentrations (25, 50, 100, 150, 200 and 250 μg/mL) to determine the IC_50_ value. The IC_50_ value is the concentration of extracts at which the cell viability was reduced to 50%. The IC_50_ value was calculated using GraphPad Prism version 5.0.5-flurouracil (5-FU) was used as a positive control drug.

### Caspase 3/7 activity assay

The assay was carried out using Apotox-Glo™ Triplex assay kit (Promega, USA), following manufacturer’s protocol. Briefly, 7 × 10^3^ cells/well/100 μL culture media were seeded at 37 °C in a 96-well white plate for 24 h. The cells were then treated with MP-HX and MP-EA for 24 h and 48 h. The concentration of the extracts used in the assay were at ~IC_50_ values for HCT116, HCC1937, HepG2 and MDA-MB-231 cells, where MP-HX concentrations were 70 μg/mL, 90 μg/mL, 75 μg/mL, and 45 μg/mL, respectively, while MP-EA concentrations were 75 μg/mL, 90 μg/mL, 130 μg/mL and 90 μg/mL, respectively. After adding the Caspase-Glo® 3/7 reagent, the cells was incubated for 30 min at room temperature and the luminescence reading was measured using Tecan Infinite® M1000 Pro multimode reader.

### Multicaspase assay

The detection of multiple caspase (caspase-1, 3, 4, 5, 6, 7, 8 and 9) activation was done using Muse™ multicaspase assay kit (Merck Millipore, USA), following the manufacturer’s instruction. Briefly, 1 × 10^5^ cells/well/1 mL culture media were seeded at 37 °C in a 12-well plate for 24 h. The cells were then treated with MP-HX and MP-EA extracts for 24 or 48 h. The concentration of the extracts used were at ~IC_50_ values for HCT116, HCC1937, HepG2 and MDA-MB-231 cells, where MP-HX concentrations were 70 μg/mL, 90 μg/mL, 75 μg/mL, and 45 μg/mL, respectively, while MP-EA concentrations were 75 μg/mL, 90 μg/mL, 130 μg/mL and 90 μg/mL, respectively. After treatment with the extracts, the cells were resuspended in 1X caspase buffer and 50 μL of the cells were transferred to 1.5 mL microcentrifuge tubes. Five μL of Muse™ multicaspase reagent working solution was added to the cells and incubated for 30 min in a 37 °C incubator. Then, 150 μL of Muse™ caspase 7-aminoactinomycin D (7-AAD) working solution was added in each tube and mixed and incubated in the dark for 5 min at room temperature. The percentage of cells with multicaspase activity was then measured using Muse™ cell analyzer (Merck Millipore, USA) flow cytometer.

### Annexin-V and dead cell assay

The assay was determined using Muse™ Annexin-V & Dead Cell (7-AAD) kit (Merck Millipore, USA), according to manufacturer’s instruction. Briefly, 1 × 10^5^ cells/well/1 mL culture media were seeded in a 12-well plate and incubated at 37 °C for 24 h. The cells were then treated with MP-HX and MP-EA extracts for 24 or 48 h. The concentration of the extracts used were at ~IC50 values for HCT116, HCC1937, HepG2 and MDA-MB-231, where MP-HX concentrations were 70 μg/mL, 90 μg/mL, 75 μg/mL, and 45 μg/mL, respectively, while MP-EA concentrations were 75 μg/mL, 90 μg/mL, 130 μg/mL and 90 μg/mL, respectively. After treatment with MP-HX and MP-EA extracts, the cells were resuspended in 1% FBS. One hundred microliter of the cells were transferred to 1.5 mL microcentrifuge tubes and 100 μL of Muse™ Annexin-V & Dead Cell reagent was added and mixed with the cells, followed by 20 min incubation at room temperature in the dark. The cells were then analyzed using Muse™ cell analyzer. The assay could identify four types of cells: i) non-apoptotic live cells: Annexin-V (−) and 7-AAD (−), ii) early apoptotic cells: Annexin-V (+) and 7-AAD (−), iii) late apoptotic cells: Annexin-V (+) and 7-AAD (+), and iv) non-apoptotic dead cells: Annexin-V (−) and 7-AAD (+).

### Caspase inhibition assay

The pan caspase inhibitor z-VAD-fmk (Biovision, USA) was used to inhibit caspases that are responsible for apoptosis induction. Briefly, 1 × 10^4^ cells/well/100 μL culture media were seeded in 96-well plate and incubated at 37 °C for 24 h. The cells were then treated with MP-HX and MP-EA extracts for 48 h in the absence and presence of z-VAD-fmk (4 μM and 8 μM). The concentration of the extracts used were at ~IC_50_ values for HCT116, HCC1937, HepG2 and MDA-MB-231 cells, where MP-HX concentrations were 70 μg/mL, 90 μg/mL, 75 μg/mL, and 45 μg/mL, respectively, while MP-EA concentrations were 75 μg/mL, 90 μg/mL, 130 μg/mL and 90 μg/mL, respectively. After 48 h, the cell viability was determined using Promega’s MTS cell viability kit, following the manufacturer’s instruction.

### Cell cycle assay

This assay was performed using Muse™ cell cycle kit (Merck Millipore, USA), according to manufacturer’s protocol. Briefly, 3 × 10^5^ cells/well/1 mL culture media were seeded in T25 culture flask and incubated at 37 °C for 24 h. The cells were then treated with MP-HX and MP-EA extracts for 24 h to measure DNA content at various stages of the cell cycle. The concentration of the extracts used were at ~IC_50_ values for HCT116, HCC1937, HepG2 and MDA-MB-231 cells, where MP-HX concentrations were 70 μg/mL, 90 μg/mL, 75 μg/mL, and 45 μg/mL, respectively, while MP-EA concentrations were 75 μg/mL, 90 μg/mL, 130 μg/mL and 90 μg/mL, respectively. The cells were fixed and washed with cold PBS and added with 200 μL of Muse™ cell cycle reagent. After 30 min of incubation at room temperature in the dark, the percentage of cells in various stages (G_o_/G_1_, S and G_2_/M) was analysed using Muse™ cell analyzer flow cytometer.

### Statistical analysis

Unless otherwise specified, each determination was done in triplicate and results were expressed as means ± standard deviation. Statistical analysis was perfomed using SPSS statistical software version 22.0 (SPSS Inc., USA). One-way analysis of variance (ANOVA) and Welch-ANOVA test were used to compare means among three or more than three groups. One-way ANOVA (when variances are equal) or Welch-ANOVA (when variances are not equal) was followed by Tukey’s or Games-Howell’s post hoc test, respectively. Values were considered to be statistically significant if the *p* value was less than 0.05. Pearson correlation were used to investigate the correlation between antioxidant components and the antioxidant activities. A correlation was considered statistically significant if the *p* value was less than 0.01 (*p* < 0.01).

## Results and Discussions

### Extraction yield

The yield for the extraction of plant materials is generally dependent on the extraction methods, the types of solvent used, as well as physical and chemical properties of plant phytochemicals [16]. In the present study, sequential extraction was employed to obtain MP leaf extracts using solvents of varying polarity. The extraction was performed sequentially using the following solvents in the order indicated: hexane (HX) > ethyl acetate (EA) > methanol (MeOH) > Water (W). This may help separate the phytochemical constituents according to their polarity. The use of solvents of varying polarity may also provide a broader coverage of extraction conditions and this could lead to higher mass transfer of plant phytochemicals.

The extraction yields of MP using various solvents are shown in Table 1. The extraction yields obtained appear to increase with increasing polarity of solvents, whereby the water extract (MP-W) showed the highest yield of 12.00%, while the hexane extract (MP-HX) had the lowest yield of 2.65%. This suggests that the extractable constituents of MP leaves are mostly polar and water soluble.

**Table 1:** Extraction yields and antioxidant components of MP

Extraction solvent	Extraction yield	Total phenolic content, TPC	Total flavonoid content, TFC
	(%)	(mg GAE/g DE)	(mg QE/g DE)
Hexane	2.65	28.39 ± 0.63^a^	36.13 ± 2.48^a^
Ethyl acetate	3.84	31.41 ± 1.07^b^	56.45 ± 1.73^b^
Methanol	5.78	61.31 ± 1.39^c^	97.85 ± 3.71^c^
Water	12.00	30.29 ± 0.75^a,b^	5.42 ± 0.27^d^

*GAE* gallic acid equivalent, *QE* quercetin equivalent, *DE* dried extract. Values are mean ± SD (*n* = 3). Means with different letters (a-d) are significantly different (*p* < 0.05)

### Total phenolic content

**Fig. 1:**
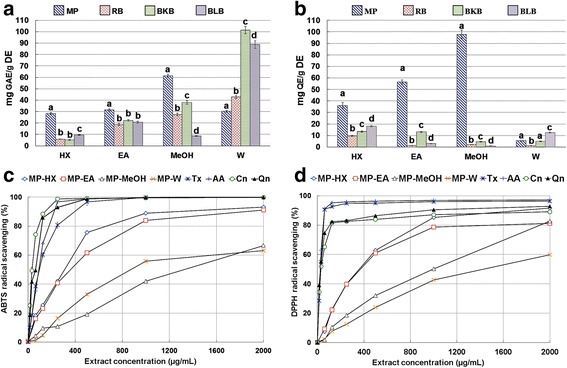
Determination of antioxidant components and in vitro antioxidant activities of MP leaf extracts. (**a**) Total phenolic content, (**b**) Total flavonoid content, (**c**) ABTS radical-scavenging, and (**d**) DPPH radical-scavenging. Values are mean ± SD (*n* = 3). RB, raspberry; BKB, blackberry; BLB, blueberry; HX, hexane; EA, ethyl acetate; MeOH, methanol; W, water. Means with different letters (a-d) are significantly different (*p* < 0.05). GAE, gallic acid equivalent; QE, quercetin equivalent; DE, dried extract; Tx, trolox; AA, ascorbic acid; Cn, catechin; Qn, quercetin

Phenolic compounds are the major constituents responsible for antioxidant activities in plants. They act as free radical scavengers, metal chelating agents and chain breaking antioxidants [17]. The TPC values of MP extracts are shown in Table 1. The varying TPC values demonstrated by the extracts suggest that MP leaves contained phenolics of varying polarities. The present study indicated that MP leaves contain predominantly polar phenolic compounds, although substantial amount of semi-polar and non-polar phenolic compounds was also detected in the leaves, as reflected by MP-HX and MP-EA TPC values. MP-MeOH showed the highest TPC value of 61.31 ± 1.39 mg GAE/g DE, followed by MP-EA, MP-W and MP-HX extracts. To gauge a comparative picture on the antioxidant potential of MP, berries fruit extracts were prepared employing the same sequential extraction protocol as of MP leaves. Interestingly, the TPC values for MP-HX, MP-EA and MP-MeOH extracts were significantly higher than raspberry, blackberry and blueberry equivalent extracts (Fig. 1A).

### Total flavonoid content

The estimation of flavonoids content in plant extracts is of significance for the assessment of its nutraceutical value, since flavonoids are phytonutrients that are renowned for their antioxidant activity. Flavonoids could prevent oxidative stress through the modulation of ROS generating enzymes activity, scavenging of free radicals and chelation of metal ions. Flavonoids also exhibit various health promoting bioactivities, which include anticancer, antimicrobial, anti-inflammatory and hepatoprotective activities [18, 19].

The total flavonoid content (TFC) value of MP extracts is presented in Table 1. Among MP extracts, MP-MeOH exhibited the highest TFC, followed by (in descending order) MP-EA, MP-HX and MP-W. For berries fruit extracts (raspberry, RB; blackberry, BKB; blueberry, BLB), the TFC values obtained were in the following descending order: BLB-HX, BKB-EA, BKB-MeOH and BLB-W extracts (Fig. 1B). Interestingly, the TFC values of MP-HX, MP-EA and MP-MeOH were notably higher than the same extracts of berries fruit. The TFC value of MP-HX was 2.0 times higher than BLB-HX, while MP-EA TFC value was 4.3 times higher than BKB-EA. The TPC value of MP-MeOH was 20.9 times higher than BKB-MeOH. However, the TPC value of MP-W was 2.3 times lower than BLB-W.

### Ferric reducing antioxidant power assay

In this assay, antioxidants can cause the reduction of the yellow-colored ferric-2,4,6-tripyridyl-s-triazine complex (Fe^3+^ − TPTZ) to the ferrous form (Fe^2+^-TPTZ), forming a blue-colored product that can be spectrophotometrically monitored at 593 nm [12]. The amount of Fe^2+^-TPTZ complex is correlated to the amount antioxidant molecules present in the extract, introduced into the reaction mixture. The FRAP value of the extracts were deduced based on FeSO_4_ standard curve (y = 0.6192× + 0.1143, R^2^ = 0.999). All MP extracts demonstrated high FRAP values, ranging from 300.65 ± 3.54 to 1150.97 ± 2.93 mmol Fe^2+^/g DE (Table 2). The highest activity was exhibited by MP-W (1150.97 ± 2.93 mmol Fe^2+^/g DE) followed by (in descending order) MP-MeOH, MP-EA and MP-HX. MP-W demonstrated notable FRAP values as compared to the positive controls, although the values were approximately 48%, 45% and 40% of that shown by catechin, gallic acid and ascorbic acid, respectively.

**Table 2:** Antioxidant activities of MP leaf extracts

Extraction solvent	FRAP(mmol Fe^2+^/g DE)	ABTS^●+^ (IC_50_ μg/mL)	DPPH^●^(IC_50_ μg/mL)	CAA (Hs27)(EC_50_ μg/mL)
Hexane	300.65 ± 3.54^a^	267.73 ± 5.58^a^	327.40 ± 3.80^a^	11.30 ± 0.68^a^
Ethyl acetate	482.23 ± 5.81^b^	322.63 ± 4.67^b^	363.60 ± 4.26^b^	37.32 ± 0.68^b^
Methanol	811.07 ± 11.02^c^	1264.33 ± 28.38^c^	838.93 ± 27.40^c^	208.94 ± 2.67^c^
Water	1150.97 ± 2.93^d^	997.73 ± 14.21^d^	1368.00 ± 28.93^d^	339.62 ± 2.07^d^
Positive controls				
Tx	-	88.84 ± 3.76^e^	26.08 ± 0.42^e^	4.02 ± 0.15^e^
AA	2889.59 ± 13.56^e^	79.64 ± 2.22^e^	22.95 ± 0.50^f^	ND
GA	2560.15 ± 28.86^f^	ND	ND	ND
Cn	2387.02 ± 46.65^f^	31.29 ± 1.11^f^	28.73 ± 0.34^g^	ND
Qn	ND	46.95 ± 0.58^g^	22.43 ± 0.58^f^	0.57 ± 0.01^f^
PP	ND	ND	ND	1.12 ± 0.08^g^

*Tx* trolox, *AA* ascorbic acid, *GA* gallic acid, *Cn* catechin, *Qn* quercetin, *PP* polyphenon-60, *DE* dried extract, *ND* not done. Values are mean ± SD (*n* = 3). The mean values in each column with different letters (a-g) are significantly different (*p* < 0.05)

### ABTS radical-scavenging assay

In this assay, the oxidation of ABTS reagent by potassium persulfate (K_2_S_2_O_8_) results in the formation of ABTS radical cations (ABTS^●+^), characterised by a blue-green colored solution. The ABTS^●+^ concentration can be monitored through spectrophotometric measurement at 734 nm [13]. A decrease in absorbance value is observed when ABTS^●+^ is scavenged by antioxidant molecules that are present in the extracts. The ABTS^●+^ scavenging activity of MP extracts are shown in Fig. 1C. At 1.0 mg/mL, MP-HX and MP-EA were able to scavenge more than 80% of ABTS radicals, whereas MP-MeOH and MP-W scavenging activity were approximately 41 and 55%, respectively. The IC_50_ values of the extracts are presented in Table 2. MP-HX showed the most notable scavenging activity, with an IC_50_ value of 267.73 ± 5.58 μg/mL, followed by (in descending order) MP-EA, MP-W and MP-MeOH. MP-HX showed notable ABTS radical-scavenging activity, although its IC_50_ values were ~3.0, 3.3, 5.7 times higher than trolox (Tx), ascorbic acid (AA) and quercetin (Qn), respectively. Similarly, MP-EA showed notable scavenging activity, although its IC_50_ values were 3.6, 4.0 and 6.8 times higher than Tx, AA and Qn, respectively.

### DPPH radical-scavenging assay

DPPH radical-scavenging assay is the most widely used in vitro antioxidant assay for evaluation of antioxidant activity. DPPH^•^ is a purple-colored radical which can be reduced by antioxidants to form a yellow-colored 2,2-diphenyl-1-picrylhydrazine, and this reaction can cause a decrease in absorbance value at 517 nm [14]. This assay is suitable for measuring radical-scavenging activity of both hydrophilic and lipophilic phytochemicals [20]. The results are shown in Table 2 and Fig. 1D. MP-HX exhibited the strongest DPPH^•^ scavenging activity, similar to that seen in ABTS^●+^ scavenging assay. Based on the IC_50_ values, the relative DPPH^•^ scavenging activity of the extracts (in descending order) were: MP-HX > MP-EA > MP-MeOH > MP-W.

### Cellular antioxidant activity assay

The cellular antioxidant activity (CAA) assay is generally considered to be more accurate in evaluating the antioxidant potential of phytochemicals or plant extract, as compared to in vitro antioxidant assays. This is because the assay utilizes a cell-based model that takes into account of the complex biological processes of a living system, such as the absorption, distribution, metabolism and bioavailability of phytochemicals. These are among the crucial factors that can greatly affect antioxidant capacity [15, 21]. In the present study, the CAA assay was carried out using the non-cancerous human fibroblast cell line, Hs27.

**Fig. 2:**
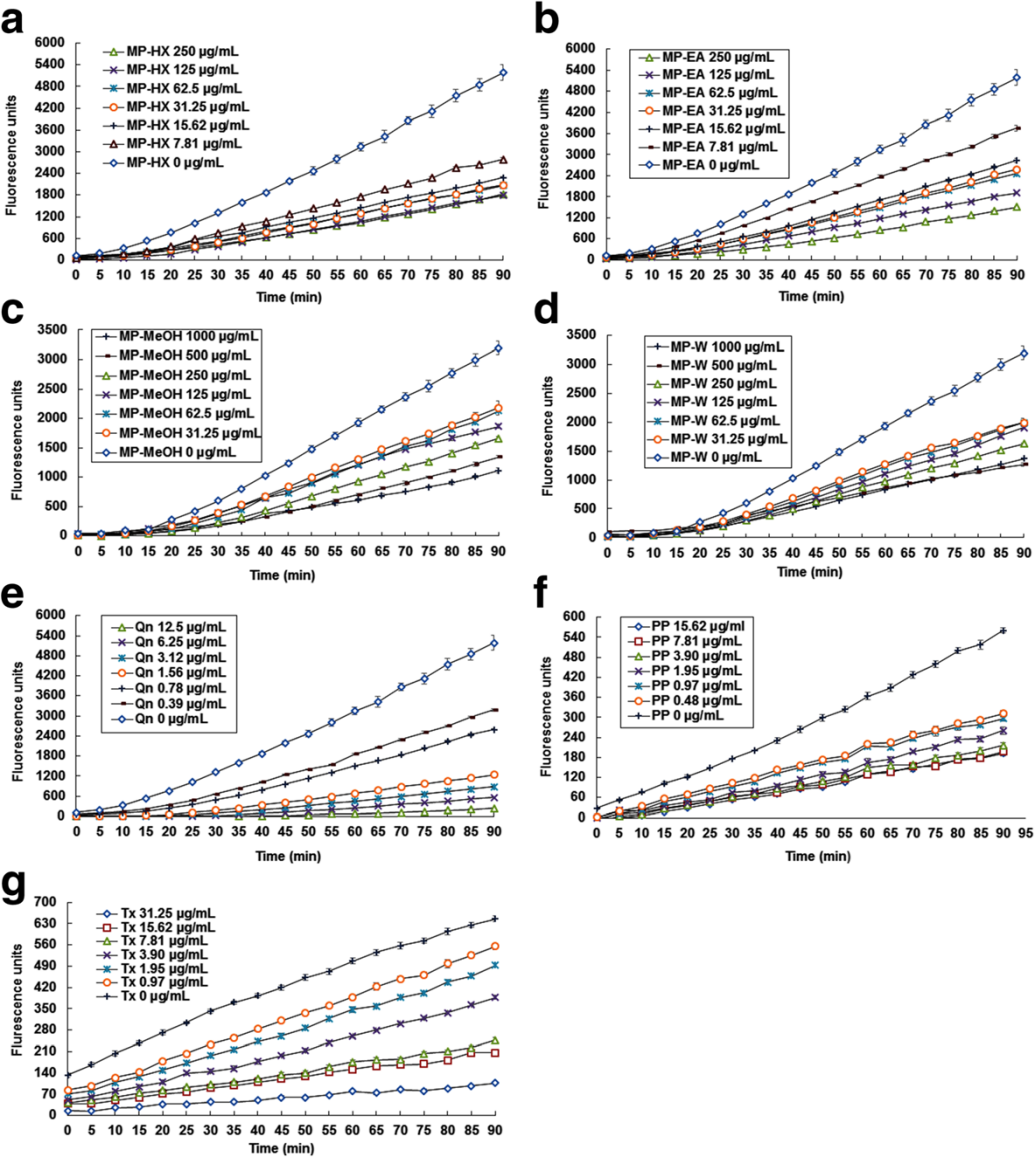
Cellular antioxidant activity of MP leaf extracts. Inhibition of peroxyl radical-induced oxidation of DCFH_2_ in Hs27 cells by **a** MP-HX, **b** MP-EA, **c** MP-MeOH, **d** MP-W, **e** Quercetin **f** Polyphenon-60 and **g** Trolox. The *curves* shown in each graph are from a single experiment (mean ± SD, *n* = 3)

**Fig. 3:**
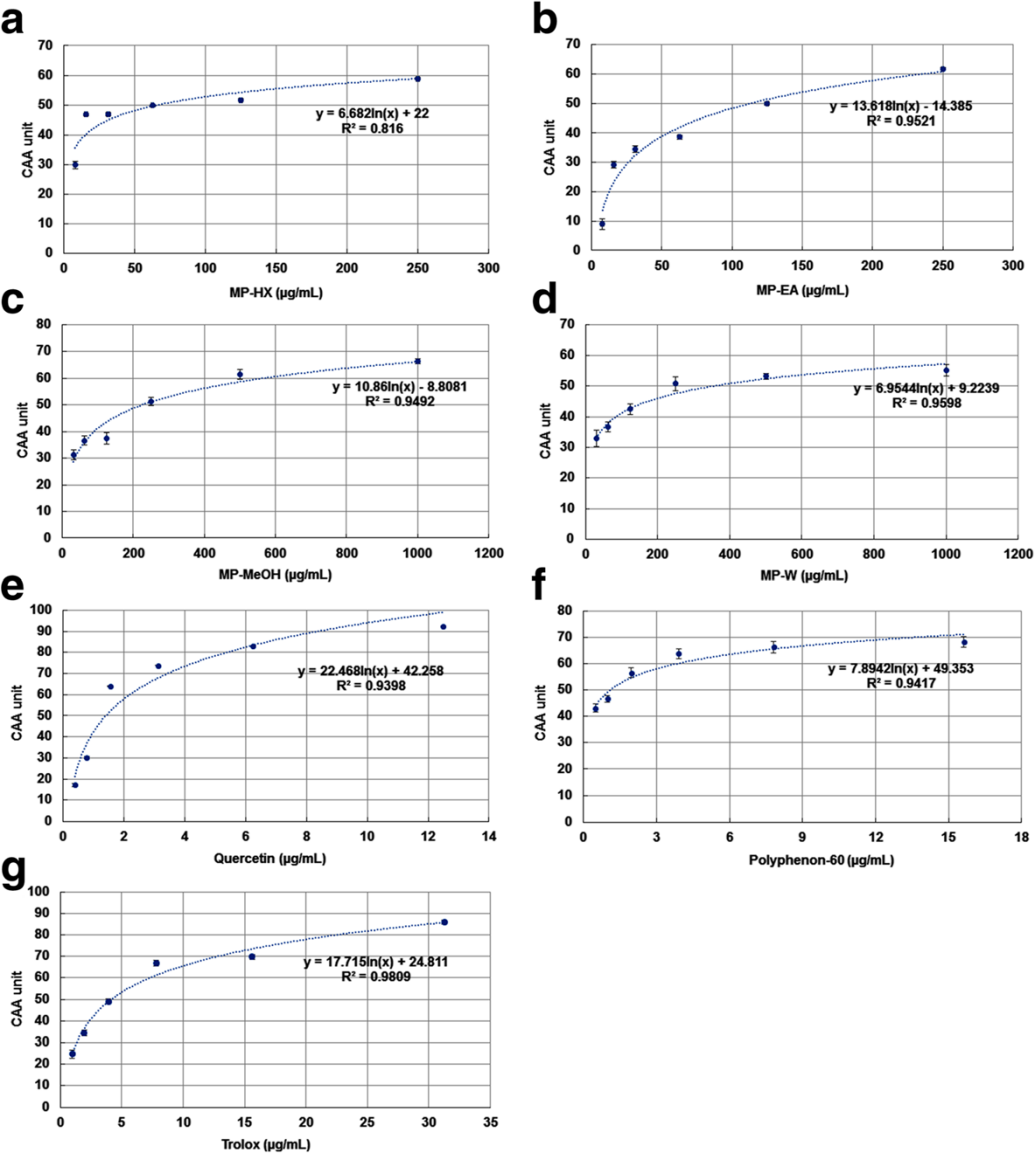
Dose-response *curve* for inhibition of peroxyl radical-induced DCFH_2_ oxidation in Hs27 cells by MP leaf extracts and positive controls. **a** MP-HX, **b** MP-EA, **c** MP-MeOH, **d** MP-W, **e** Quercetin, **f** Polyphenon-60 and **g** Trolox. The *curves* shown in each graph are from a single experiment (mean ± SD, *n* = 3)

The results showed that the pure phytochemical compounds (positive controls) and MP extracts were able to inhibit the oxidation of DCFH_2_ by ABAP-generated peroxyl radicals in a dose-dependent manner (Fig. 2). The median effective dose (EC_50_) was determined from the median effect plot (Additional file 1: Figure S2). The EC_50_ value of quercetin in Hs27 cell line was 1.88 ± 0.03 μM, and this value is 2.7 times lower than that observed in HepG2 cells, which was 5.09 ± 0.19 μM [18]. This difference may have been due to the fact that HepG2, being a type of cancer cell line, experienced higher oxidative stress environment compared to non-cancerous Hs27 cell line. As for MP extracts, all of them showed notable cellular antioxidant activity (Fig. 3). Based on the EC_50_ values (Table 2), MP-HX showed the most notable CAA value, followed by MP-EA. However, MP-HX and MP-EA showed lower activities compared to trolox, with EC_50_ values that were 2.8 and 9.3 folds higher than trolox, respectively. MP-MeOH and MP-W exhibited lower antioxidant potential, since their EC_50_ values were significantly higher than MP-HX and MP-EA. In comparison to common fruits and vegetables, the findings in the present study indicated that the MP extracts exhibited a more pronounced cellular antioxidant activity. The EC_50_ value of MP extracts in Hs27 were ~31 to 956 times lower than blueberry (EC_50_ in HepG2 = 10.81 ± 0.44 mg/mL), and they were ~338 to 10,176 lower than broccoli (EC_50_ in HepG2 = 115 ± 15 mg/mL) [15, 22].

### Correlation analysis

Various antioxidant assays have been reported in the literature, which include in vitro and CAA antioxidant assays. These assays are routinely used to evaluate antioxidant potential of plant extracts, but they do not necessarily provide consistent picture when compared against each other, due to differences in their mechanism of assessing antioxidant activity. Comparing the value of antioxidant activities of MP extracts as indicated by the different assays, there was a strong to very strong positive correlation between ABTS, DPPH and CAA assays, ranging from 0.777 to 0.993 (Table 3), suggesting that these assays could provide a consistent picture of antioxidant activity of MP extracts. A correlation analysis between TPC and TFC with antioxidant activity was also performed, to evaluate their relationship. A strong positive correlation of 0.849 was observed between TFC and TPC, suggesting that flavonoids are the predominant phenolic compounds in MP. Although TPC demonstrated a positive correlation (0.750) with ABTS, its correlation with DPPH and CAA was weak. In contrast, the correlation of TFC with ABTS was weak, and negative correlation between TFC with DPPH and CAA assays were also observed. These observations suggest that polyphenols may not be the major constituents that are responsible for the antioxidant activity of MP. Weak correlation between polyphenols content and antioxidant activity have also been reported in commonly consumed fruits [23].

**Table 3:** Pearson correlation analysis for antioxidant components and activities of MP leaf extracts

	TPC	TFC	ABTS	DPPH	CAA
TPC	1	0.849*	0.750*	0.171	0.273
TFC	0.849*	1	0.302	−0.351	–0.247
ABTS	0.750*	0.302	1	0.777*	0.838*
DPPH	0.171	−0.351	0.777*	1	0.993*
CAA	0.273	−0.247	0.838*	0.993*	1

*Significant correlation at *p* < 0.01

### Anti-proliferative activity of MP leaf extracts

#### Cell viability assay

The anti-proliferative activities of the extracts were initially evaluated against the cancer cell lines at 250 μg/mL, using Promega MTS cell viability kit. The result indicated that MP-HX and MP-EA were able to reduce the viability of all cancer lines tested to about 30%, indicating their promising anti-proliferative activity (Additional file 2: Figure S1). MP-MeOH and MP-W did not demonstrate notable anti-proliferative activity. They exerted modest inhibition of HCT116 proliferation at 250 μg/mL, reducing viability to 73.8% and 78.7%, respectively.

**Fig. 4:**
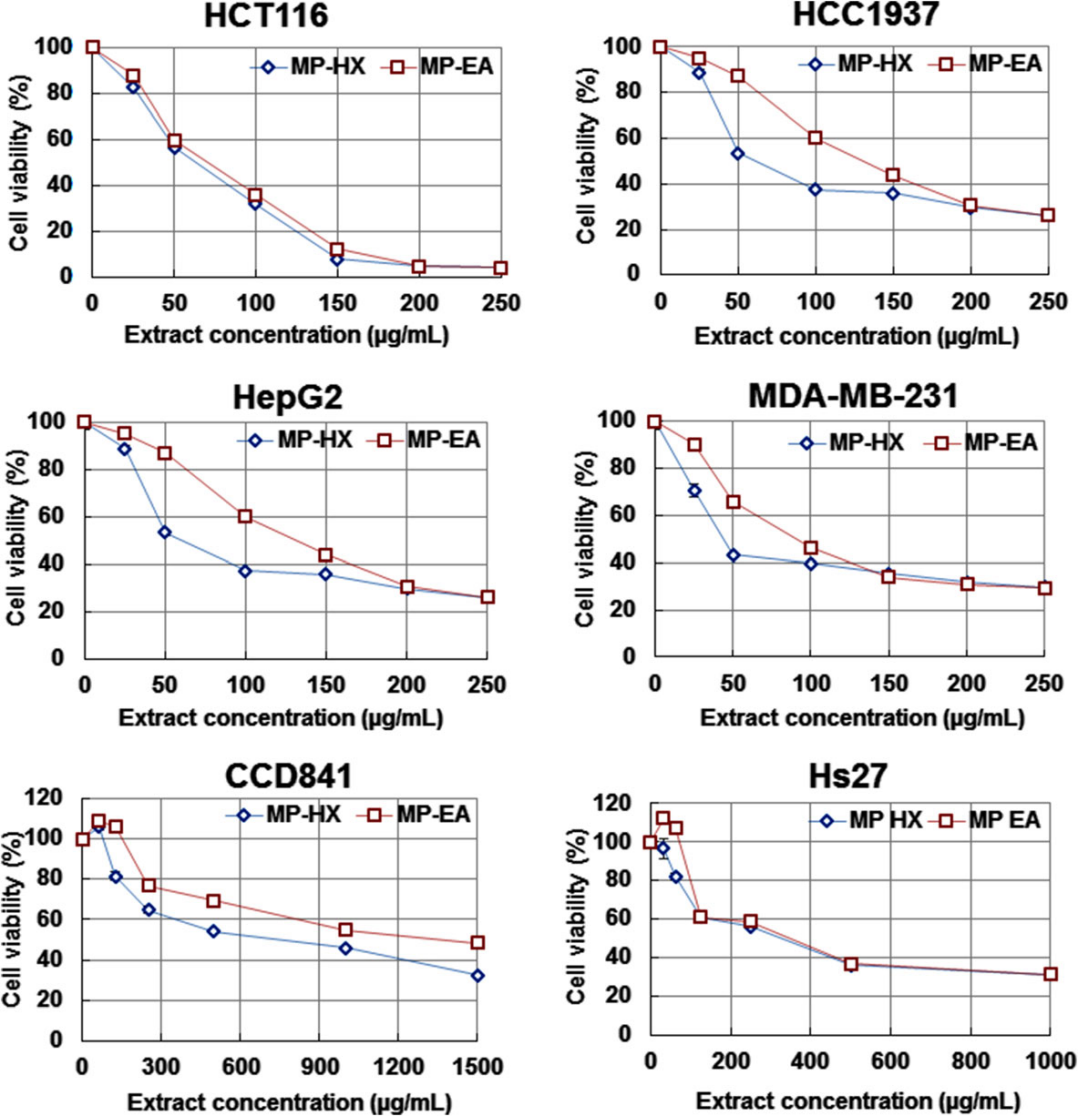
MTS cell viability assay. Dose response *curves* for the cytotoxic effect of MP-HX and MP-EA (48 h treatment) on cancerous (HCT116, HCC1937, HepG2, MDA-MB-231) and non-cancerous (CCD841, Hs27) cell lines

The IC_50_ values of MP-HX and MP-EA against the cancer cell lines were then investigated further. A dose-dependent anti-proliferative activity was observed for MP-HX and MP-EA extracts on HCT116, HCC1937, HepG2 and MDA-MB-231 cell lines (Fig. 4). MP-HX exhibited promising anti-proliferative activity against the entire cancer cell lines tested, with IC_50_ concentrations ranging from 58.04 ± 0.96 to 94.80 ± 3.01 μg/mL (Table 4). MP-EA also exhibited potent anti-proliferative activity against HCT116, HCC1937 and MDA-MB-231, with IC_50_ values ranging from 64.69 ± 0.72 to 97.09 ± 1.10 μg/mL (Table 4). Interestingly, MP-HX IC_50_ value on HepG2 cells was lower than that shown by 5-FU.

**Table 4:** Cell viability assay. IC_50_ values of MP-HX and MP-EA on cancer and non-cancerous cell lines

Treatment	HCT116	HCC1937	HepG2	MDA-MB-231	CCD841	Hs27
	IC_50_ (μg/mL)	IC_50_ (μg/mL)	IC_50_ (μg/mL)	IC_50_ (μg/mL)	IC_50_ (μg/mL)	IC_50_ (μg/mL)
MP-HX	58.04 ± 0.96^a^	94.80 ± 3.01^a^	79.41 ± 1.88^a^	57.81 ± 3.49^a^	680.93 ± 8.76^a^	311.30 ± 12.41^a^
MP-EA	64.69 ± 0.72^b^	95.71 ± 0.36^a^	130.93 ± 3.17^b^	97.09 ± 1.10^b^	1218.33 ± 5.50^b^	357.13 ± 5.11^b^
5-FU	13.81 ± 0.58^c^	79.02 ± 2.12^b^	95.02 ± 3.56^c^	24.45 ± 4.52^c^	9.64 ± 0.02^c^	> 250

Values are mean ± SD (*n* = 3). Mean values in each column with different letters (a, b and c) are significantly different (*p* < 0.05)

To evaluate the selectivity and toxicity of MP-HX and MP-EA against non-cancerous cell lines, they were tested against CCD841 and Hs27 cell lines, which were normal colon and fibroblast cell lines, respectively. Both extracts showed relatively higher IC_50_ values on these cell lines (ranging from 311.30 ± 12.41 to 1218.33 ± 5.50 μg/mL) (Table 4), suggesting the extracts were selectively more toxic towards the cancer cell lines.

#### Induction of apoptosis by MP-HX and MP-EA

Apoptosis is a form of programmed cell death with essential roles in removal of damaged or abnormal cells from the body. Apoptosis can be induced through the intrinsic (mitochondrial) or extrinsic (death receptor) pathway. It is characterized by morphological and cellular changes which include membrane blebbing, phosphatidylserine (PS) extrusion [24], cellular shrinkage and DNA fragmentation [25]. Perturbation of apoptosis could arise from overexpression of anti-apoptotic and/or down regulation of pro-apoptotic proteins, and such scenarios could lead to the development of chronic conditions such as cancer and neurodegenerative diseases [26]. In many type of human cancers, apoptosis is often dysregulated. Thus, when screening for new anticancer drugs, the candidate drug should ideally demonstrate selective cytotoxicity, by its preferential induction of apoptosis in cancer cells with minimal toxicity on non-cancerous or normal cells [27]. In the present study, the effect of MP-HX and MP-EA on apoptosis was evaluated through four different assays, which include measurements of caspase 3/7 activity, multicaspase activity, caspase enzyme inhibition and Annexin-V/7-AAD staining.

#### Caspase-3/7 assay

Caspases (cysteinyl aspartases) are a family of proteolytic enzymes which play a key role in the induction of apoptosis [28]. They are classified as initiator caspases (−2, −8, −9. -10) and executioner caspases (−3, −6, −7) [29]. Initiator caspases can be activated by the intrinsic or extrinsic pathways, which themselves can activate the executioner caspases and leading to apoptosis induction. Activation of caspase-3/7 is an important biomarker for the detection of apoptosis [29].

**Fig. 5:**
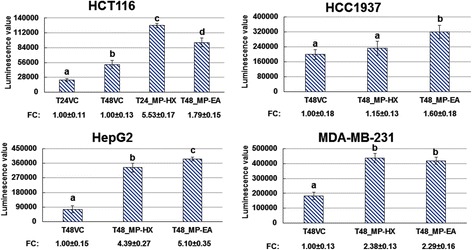
The effect of MP-HX or MP-EA on caspase 3/7 activity in cancer cell lines. The bar charts depict luminescence value for caspase 3/7 activity. The concentration of the extracts used in each treatment was at ~IC_50_ value for the corresponding cell lines indicated. T24/T48, 24 or 48 h treatment, respectively. VC, vehicle control; FC, fold change compared to VC. Values are mean ± SD (*n* = 3). The values with different letters (a-d) are significantly different, *p* < 0.05

Caspase-3/7 assay was done using Promega Apotox-Glo™ assay kit which included a luminogenic caspase-3/7 substrate. An increase in luminescence value indicates caspase-3/7 activation. The results indicated that both MP-HX and MP-EA extracts were able to significantly (*p* < 0.05) activate caspase-3/7 activity in almost all of the cancer cell lines tested (Fig. 5). The cancer cells treated with MP-HX showed caspase-3/7 activation in MDA-MB-231, HepG2 and HCT116 that was respectively 2.38, 4.39 and 5.53 folds higher, compared to the corresponding vehicle control. MP-HX apparently did not induce caspase-3/7 activation in HCC1937 for the 48 h treatment. MP-EA treatment induced caspase-3/7 activation in HCC1937, HCT116, MDA-MB-231 and HepG2, that was respectively 1.60, 1.79, 2.29 and 5.10 folds higher, compared to the corresponding vehicle control.

#### Multicaspase assay

**Fig. 6:**
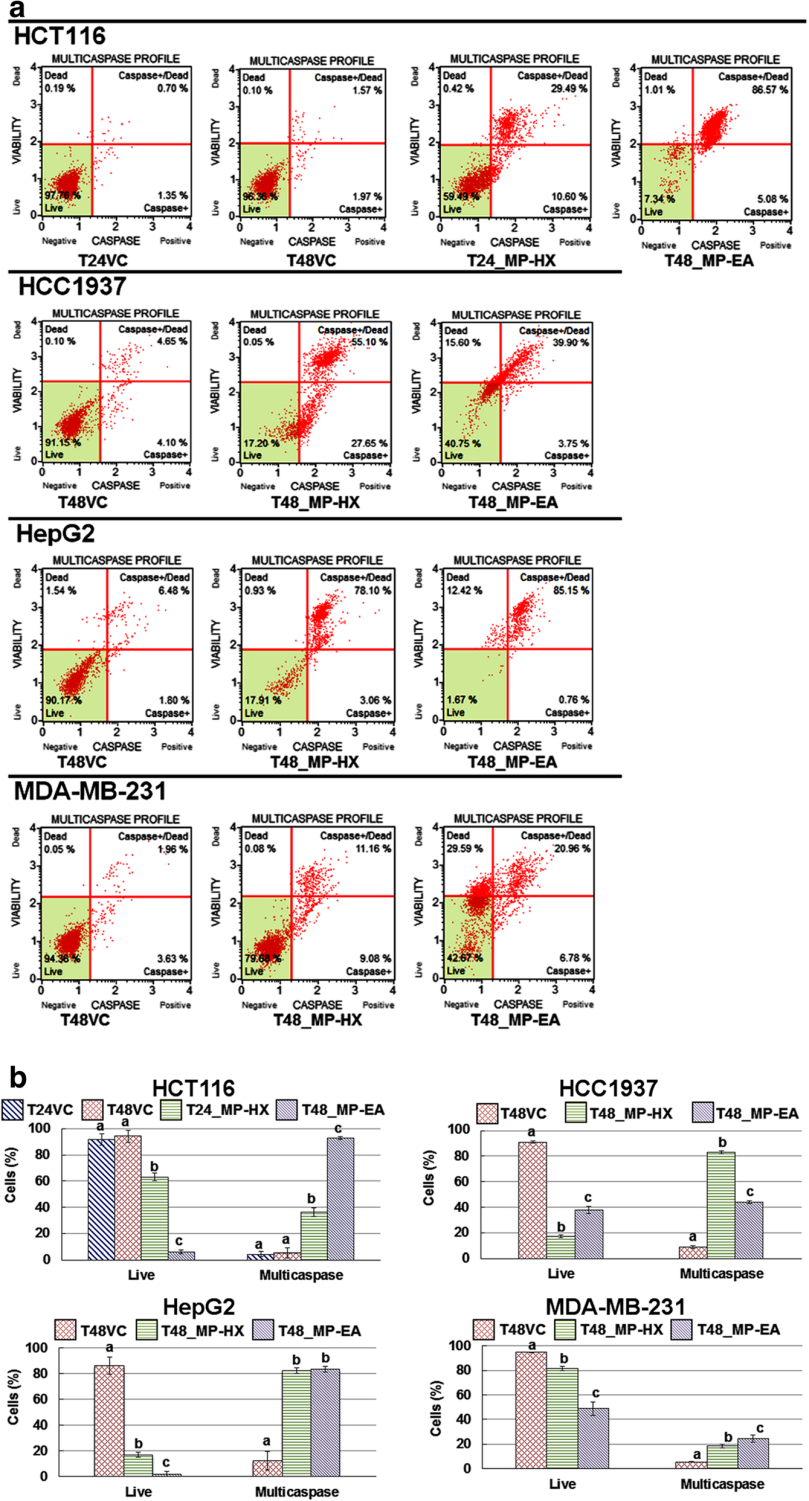
Cell viability and multicaspase enzyme activation assays. (**a**) The plots depict the effect of MP-HX and MP-EA treatments in the cancer cell lines indicated. Each plot is a representative figure of the three replicates of each determination. (**b**) The bar charts depict the percentage of live cells and those with multicaspase enzyme activation. The concentration of the extracts used in each treatment was at ~IC_50_ value for the corresponding cell lines indicated. T24/T48, 24 or 48 h treatment, respectively. VC, vehicle control. Values are mean ± SD (*n* = 3). The values with different letters (a-d) are significantly different, *p* < 0.05

Muse™ multicaspase (Merck Millipore, USA) assay kit was used to detect initiator as well as executioner caspases activation (caspase-1, 3, 4, 5, 6, 7, 8, and 9). The results indicated that both MP-HX and MP-EA were able to significantly increase the percentage of cells with activated caspases in the entire cancer cell lines tested **(**Fig. 6). MP-HX and MP-EA treatments on HCT116 significantly increased the percentage of cells with caspases activation, whereby the percentage of cells were 8.2 and 17.4 folds higher than the corresponding vehicle control, respectively. MP-HX and MP-EA treatments on HCC1937 significantly increased the percentage of cells with caspases activation, whereby the percentage of cells were 9.4 and 5.0 folds higher than the corresponding vehicle control, respectively. For HepG2 cell line, MP-HX and MP-EA treatments significantly increased the percentage of cells with caspases activation, whereby the percentage of cells were 6.5 and 6.6 folds higher than the corresponding vehicle control, respectively. For MDA-MB-231 cell line, MP-HX and MP-EA treatment significantly increased the percentage of cells with caspases activation, whereby the percentage of cells with caspases activation were 3.5 and 4.7 folds higher than the corresponding vehicle control, respectively. Taken together, caspase-3/7 and multicaspase assays result suggest that MP-HX and MP-EA were able to induce caspase-dependent apoptotic cell death in the entire cancer cell lines tested.

#### Annexin-V and dead cell assay

Phosphatidylserine is a component of a cell membrane that is normally restricted in the inner leaflet of the membrane. During the early stage of apoptosis, PS is extruded to the outer leaflet of the membrane [24]. Annexin-V is a calcium dependent phospholipid binding protein with a high binding affinity towards PS. The event of annexin-V binding with the PS molecules that are extruded in early apoptotic cells can be detected by flow cytometric analysis. The 7-AAD dye is used to detect the cell membrane structural integrity, as the dye is only permeable to the membrane of dead cells, but impermeable to live and healthy cells, as well as early apoptotic cells.

**Fig. 7:**
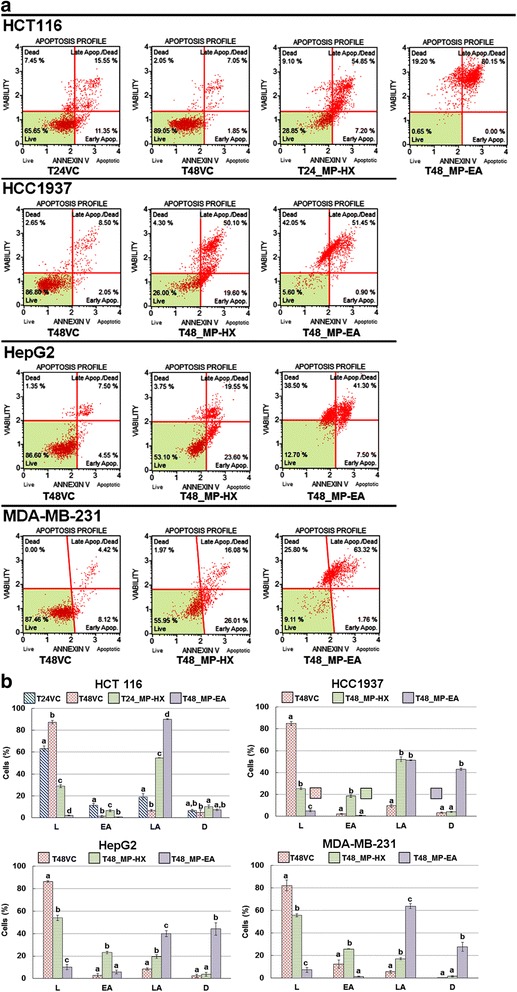
Annexin-V & Dead Cell (7-AAD) flow cytometry analysis. Apoptotic effect of MP-HX and MP-EA on HCT116, HCC1937, HepG2 and MDA-MB-231 cell lines. (**a**) Apoptosis profile plot. Each plot is a representative figure of the three replicates of each determination. (**b**) Bar charts depicting percentage of live, dead and apoptotic cells for the treatments on the corresponding cell lines. Values are mean ± SD (*n* = 3). The values with different letters (a-d) are significantly different, *p* < 0.05. The concentration of the extracts used in each treatment was at ~IC_50_ value for the corresponding cell lines indicated. T24, 24 h treatment; T48, 48 h treatment; VC, vehicle control. L, live cells; EA, early apoptotic cells, LA, late apoptotic cells; D, dead cells

The results of the present study indicated that both MP-HX and MP-EA extracts were cytotoxic to the entire cancer cell lines tested, being able to significantly reduce viability of the cancer cells (Fig. 7). In the HCT116 cells, treatments with MP-HX and MP-EA significantly reduced cell viability to 28.81 ± 1.30 and 2.03 ± 0.18%, respectively. The treatments with MP-HX and MP-EA also markedly increased the percentage of late apoptotic cells in HCT116, which were 54.75 ± 0.10 and 90.23 ± 0.59%, respectively, compared to the corresponding vehicle controls, which were 18.95 ± 2.94 and 6.30 ± 1.06%, respectively. For the HCC1937 cells, treatments with MP-HX and MP-EA significantly reduced cell viability to 25.31 ± 0.89 and 4.90 ± 0.85%, respectively, compared to the corresponding vehicle control, which was 84.98 ± 1.61%. MP-HX also markedly increased the percentage of early apoptotic cells in HCC1937 to 18.50 ± 1.31%, compared to the corresponding vehicle control, which was only 2.21 ± 0.42%. The treatments with MP-HX and MP-EA also increased the percentage of late apoptotic cells in HCC1937, which were 52.13 ± 2.15 and 51.33 ± 0.43%, respectively, compared to the corresponding vehicle control, which was only 9.53 ± 1.07%.

For the HepG2 cells, treatments with MP-HX and MP-EA significantly reduced cell viability to 53.88 ± 2.37 and 10.11 ± 2.44%, respectively, compared to the corresponding vehicle control, which was 86.30 ± 0.74%. MP-HX also markedly increased the percentage of early apoptotic cells in HepG2 to 23.10 ± 1.18%, compared to the corresponding vehicle control, which was only 2.93 ± 1.40%. The treatments with MP-HX and MP-EA also increased the percentage of late apoptotic cells in HepG2, which were 19.38 ± 1.71 and 39.88 ± 2.54%, respectively, compared to the corresponding vehicle control, which was only 8.43 ± 1.00%.

For the MDA-MB-231 cells, treatments with MP-HX and MP-EA significantly reduced cell viability to 55.72 ± 1.28 and 7.17 ± 2.29%, respectively, compared to the corresponding vehicle control, which was 82.14 ± 4.78%. MP-HX also markedly increased the percentage of early apoptotic cells in MDA-MB-231 to 25.79 ± 0.18%, compared to the corresponding vehicle control, which was only 12.29 ± 3.64%. The treatments with MP-HX and MP-EA also increased the percentage of late apoptotic cells in MDA-MB-231, which were 16.97 ± 1.12 and 63.81 ± 2.21%, respectively, compared to the corresponding vehicle control, which was only 5.52 ± 1.30%.

#### Pan caspase inhibition assay

**Fig. 8:**
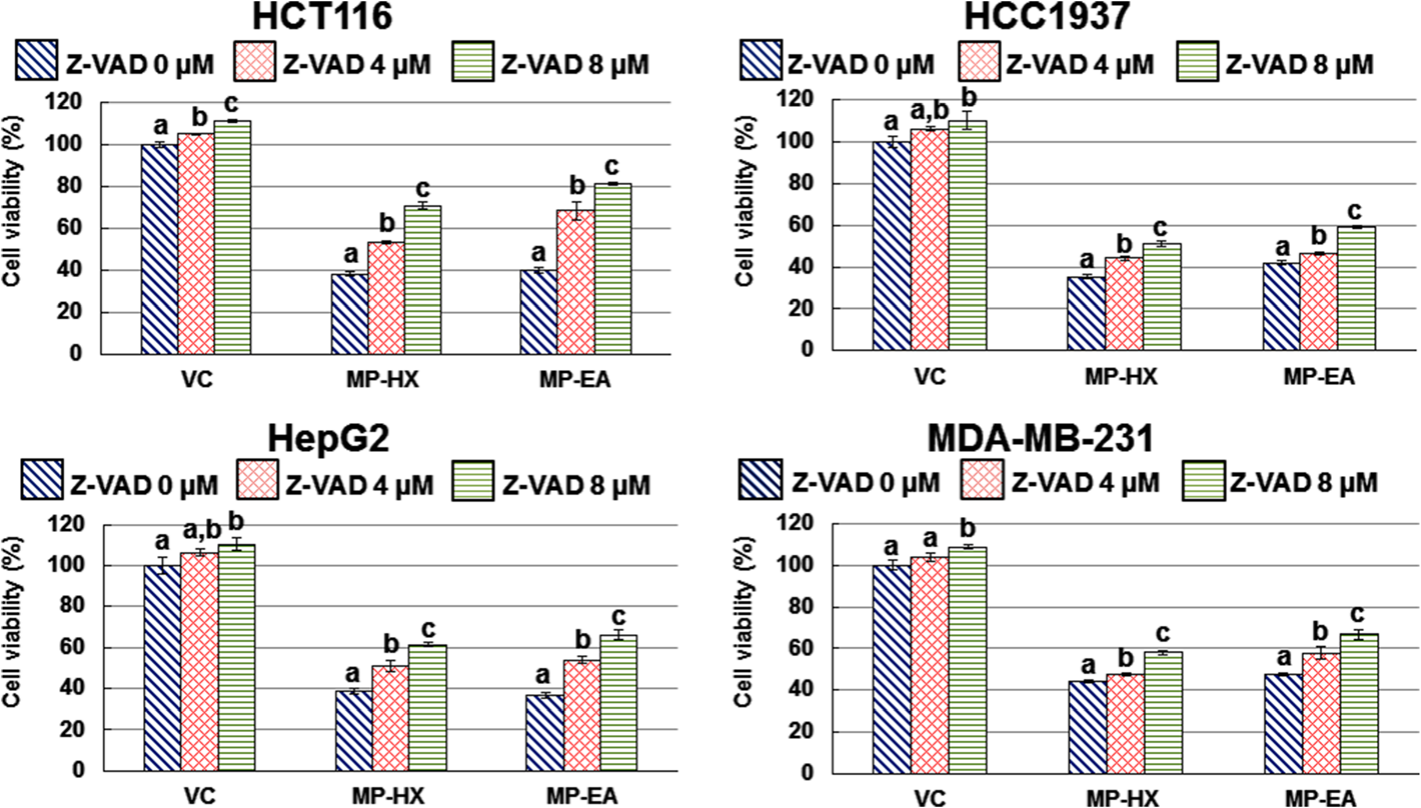
Pan caspase inhibitor assay. MTS cell viability assay (48 h) was employed using MP-HX or MP-EA extracts on the cell lines indicated, in the presence or absence of Z-VAD-FMK inhibitor. The concentration of the extracts used in each treatment was at ~IC_50_ value for the corresponding cell lines indicated. Values are mean ± SD (*n* = 3). The values with different letters (a-c) are significantly different, *p* < 0.05

This assay was performed to independently confirm that apoptosis induction of MP-HX and MP-EA extracts in the cancer cell lines was caspase dependent. The z-VAD-FMK (carbobenzoxy-valyl-alanyl-aspartyl-[O-methyl]-fluoromethylketone) reagent is a cell-permeant pan caspase inhibitor that irreversibly binds to the catalytic site of caspase proteases, preventing apoptosis induction. The effect of the inhibitor on the viability of the cancer cells in the presence and absence of MP-HX and MP-EA extracts are shown in Fig. 8. The results indicated that z-VAD-FMK inhibitor (at 4 and 8 μM) was generally non-toxic to all of the cancer cell lines. As expected, treatments of all the cancer cell lines with MP-HX or MP-EA reduced cell viability. However, the viability was significantly and dose-dependently higher when z-VAD-FMK was added in the assay. This observation suggested that the cytotoxicity induced by the extracts was due to induction of apoptosis that was caspase dependent.

#### Cell cycle analysis

The cell cycle is composed of Go, G_1_, S, G_2_ and M phases and a dysregulation in cell cycle is often observed in cancer development [30]. Chemotherapeutic drugs may exert anticancer effect through the inhibition of cancer cell proliferation by disrupting selected phases of the cell cycle [31].

**Fig. 9:**
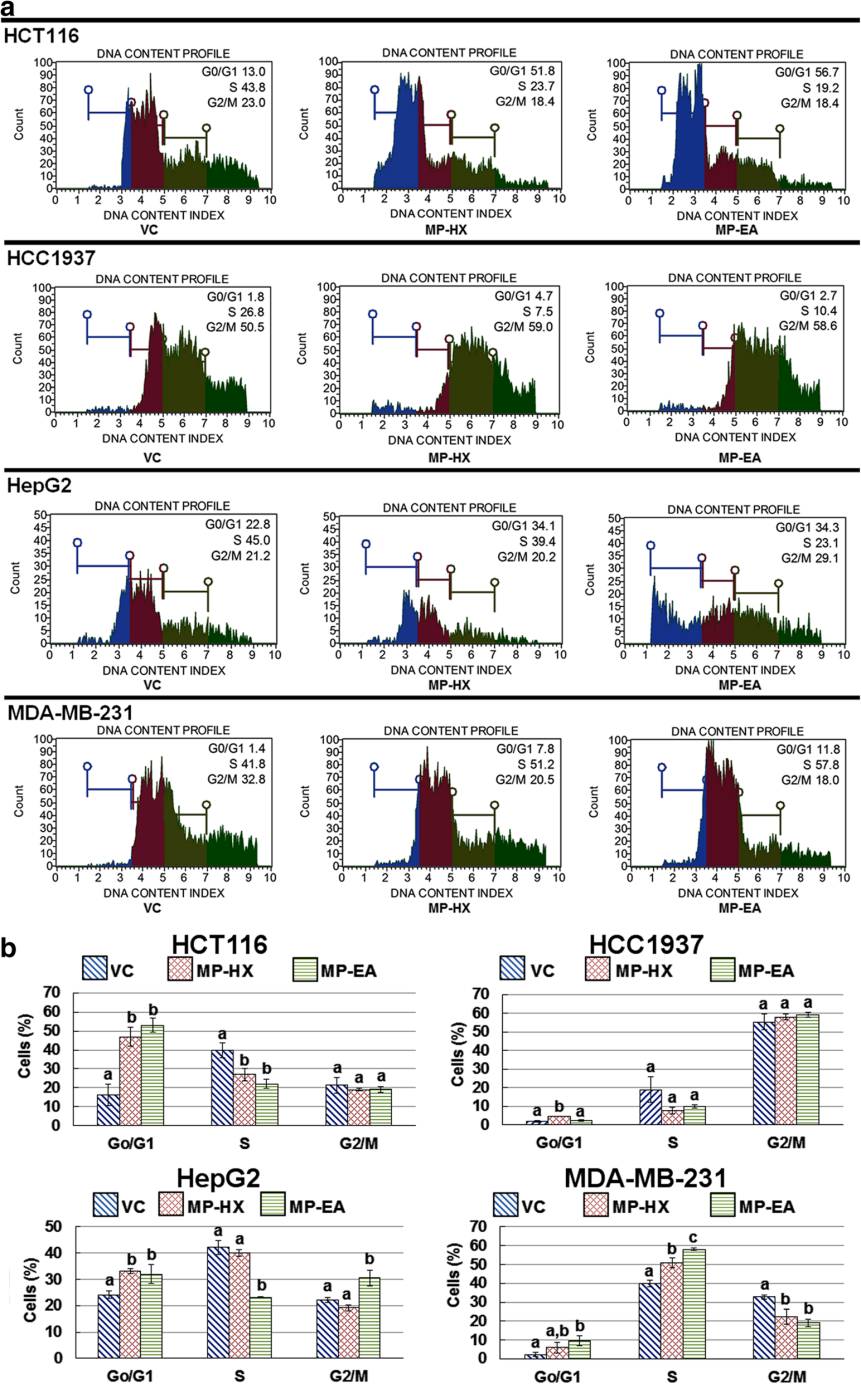
Effect of MP-EA and MP-HX on HCT116, HCC1937, HepG2 and MDA-MB-231 cell cycle distribution. The cells were treated for 24 h and the concentration of the extracts in each treatment was at ~IC_50_ value of the corresponding cell lines indicated. (**a**) Plot for DNA content profile. Each plot is a representative figure of the three replicates of each determination. (**b**) Bar charts depict the percentage of cells in the various stages of the cell cycle. Values are mean ± SD (*n* = 3). The values with different letters (a-c) are significantly different, *p* < 0.05

In the present study, flow cytometric analysis was carried out using Muse™ cell cycle kit. The flow cytometric assay profiles the percentages of cells in G_o_/G_1,_ S and G_2_/M phases of the cell cycle. The cell cycle assay results are shown in Fig. 9. In HCT116 cells, MP-HX and MP-EA significantly increased the percentage of cells in G_o_/G_1_, which were 30.6 and 36.6% higher than the control, respectively. MP-HX and MP-EA also significantly reduced the percentage of HCT116 cells in S phase, which were 12.9 and 17.9% lower than the control, respectively. Compared to the control, treatments of MP-HX and MP-EA on HCC1937 cells did not seem to significantly alter the percentages of cells in different phases of the cell cycle, except for MP-HX, whereby a small but statistically significant 2.6% increase in G_o_/G_1_ cell population was observed. It is probable that the 24 h treatment in HCC1937 did not provide an ideal window of time-frame, to demonstrate the effect of the extracts on HCC1937 cell cycle. In comparison to the control, MP-HX treatment on HepG2 caused a significant increase (+9.0%) in the percentage of cells in G_o_/G_1,_ without altering the percentages of cells in S and G_2_/M phases. In comparison to the control, the treatment of HepG2 with MP-EA resulted to 7.8% and 8.3% increase in G_o_/G_1_ and G_2_/M cells population, respectively, coupled with a 19.0% reduction of S phase cells population, suggesting that MP-EA ability to disrupt HepG2 cell cycle at multiple phases. Treatment of MDA-MB-231 with MP-HX resulted to 11.1% increase in S cells population, but a 10.3% decrease in the percentage G_2_/M cells population was also observed. This may have been sufficient to disrupt MDA-MB-231 cell cycle, resulting to MP-HX cytotoxic effect on the cells as observed earlier (Fig. 6 and Table 4). Although treatment of MDA-MB-231 with MP-EA significantly increased the percentage of cells in the S phase (+18.3%), the extract was likely to cause disruption of the MDA-MB-231 cell cycle, as there was a significant increase G_o_/G_1_ (+7.5%) cells population, coupled with a significant decrease in G_2_/M (−13.6%) cells population.

#### Cytotoxicity and apoptosis induction activities of MP-HX and MP-EA

The present study investigated the anticancer potential of MP extracts towards renowned ATCC cancer cell lines, namely HCT116 (colon cancer), HCC 1937, MDA-MB-231 (breast cancers) and HepG2 (liver cancer). The cell viability assay results indicate that MP-HX and MP-EA contain phytochemicals that are cytotoxic towards the entire cancer cell lines tested (Fig.4). These extracts showed promising anti-proliferative activity; as their IC_50_ values were less than 100 μg/mL in almost all of the cancer cell lines tested (Table 4), except for MP-EA in HepG2 cells, with an IC_50_ value of about 131 μg/mL. MP-HX and MP-EA were also selectively more cytotoxic towards the cancer cells, as they showed higher IC_50_ values (>300 μg/mL) towards non-cancerous cell lines CCD841 and Hs27. The notable anti-proliferative activity demonstrated by MP-HX and MP-EA against the four cancer cell lines prompted us to evaluate their apoptosis induction ability.

In the present study, the ability of MP-HX and MP-EA to induce apoptosis was evaluated through four different assays, which include measurements of caspase 3/7 activity, multicaspase activity, caspase enzyme inhibition and annexin-V/7-AAD staining. Overall, the results of these assays indicate that MP-HX and MP-EA were able to induce apoptosis in all of the cancer cell lines tested. Both extracts were able to induce activation of multiple caspases in all of the cancer cell lines (Figs. 5 and 6). The extracts were also able to increase the percentage of apoptotic cells as revealed by annexin-V/7-AAD flow cytometry assay (Fig.7). The apoptosis induction was also confirmed through pan caspase inhibitor (z-VAD-FMK) assay. The inhibitor was able to reduce the cytotoxicity of MP-HX and MP-EA in all of the cancer cell lines tested, in a dose-dependent manner, validating their apoptosis induction ability (Fig.8). The cell cycle assay results also suggest that MP-EA and MP-HX had the potential to disrupt cell cycle progression in three of the four cancer cell lines (Fig.9).

The present study is the first to demonstrate anticancer potential of MP. To date, none of the previous studies on MP have reported (or evaluated) its anticancer potential. Previous bioactivity studies have either used polar (eg. ethanol and methanol) MP extracts [5–8] or pure phytochemical compound (tHGA), which was isolated from methanol extract of MP [32–34]. Thus, the lack of report on MP anticancer activity may have been due to the fact that the non-polar extracts bioactivity have not been studied. In 2011, Shaari et al. reported identification of phytochemical compounds from the methanol extract of MP, which include lupeol, oleanolic acid, kokusaginine and genistein [35]. These compounds have been reported to exhibit anticancer activity [36–38]. In the present study, although MP-MeOH was observed to show the highest TPC and TFC values, the antioxidant activity was not correlated to polyphenols content. The same scenario was observed with regard to the extracts anti-proliferative activity - although the polyphenols contents of MP-HX and MP-EA were lower than MP-MeOH, they both showed higher anti-proliferative activity compared to MP-MeOH. These observations do not suggest polyphenols to be the phytochemicals that contributed MP-HX and MP-EA bioactivities. Weak correlation between total phenolic contents and anti-proliferative activities of extracts from common fruits have also been reported [39]. There are numerous class of phytochemical compounds in the plant kingdom, which include alkaloids [40], phytosterols, terpenes and carotenoids [36, 41]. Further research is warranted, to isolate and characterise the bioactive phytochemical compound(s) from MP that is/are responsible for the antioxidant and anti-proliferative activities.

## Conclusions

The findings of the present study project MP as a contain phytochemical compounds with notable antioxidant and anti-cancer activities. It is the first to report on anti-proliferative and apoptosis induction activities of MP extracts on human colorectal (HCT116), breast (HCC1937, MDA-MB-231) and hepatocellular carcinoma (HepG2) cell lines. Results of the cell viability assay indicated that MP-HX and MP-EA extracts exhibited promising anti-proliferative activity against the entire cancer lines tested, whereby the IC_50_ values obtained were mostly below 100 μg/mL. The cytotoxicity effect of MP-HX and MP-EA appeared to be selective towards the cancer cells, as the IC_50_ values of the extracts on normal colon and fibroblast cell lines (CCD841 and Hs27) were significantly higher (> 300 μg/mL) than those of the cancer cells. MP-HX and MP-EA also demonstrated notable activity to disrupt cell cycle phases in three of the four cancer cell lines. These findings suggest MP to be promising source of novel phytochemical(s) with health promoting benefits that are also valuable for nutraceutical industry and cancer therapy.

## Additional files

Additional file 1: Figure S2. Median effect plots for inhibition of peroxyl radical-induced DCFH_2_ oxidation in Hs27 cells by (A) MP-HX, (B) MP-EA, (C) MP-MeOH, (D) MP-W, (E) Quercetin, (F) Polyphenon-60 and (G) Trolox. The curves shown in each graph are from a single experiment (mean ± SD, *n* = 3). (TIFF 228 kb)

Additional file 2: Figure S1. High dose MTS cell viability assay. Effect of MP leaf extracts (250 μg/mL, 48 h) on the cell viability of HCT116, HCC1937 and HepG2 and MDA-MB-231 cell lines. Values are mean ± SD (*n* = 3). The values with different letters (a-c) are significantly different, *p* < 0.05. (TIFF 65 kb)

## Abbreviations

5-FU: Fluorouracil
7-AAD: 7-aminoactinomycin D
AA: Ascorbic acid
ABAP: 2,2′-azobis (2-amidinopropane) dihydrochloride
ABTS: 2,2′-azinobis-(3-ethylbenzothiazoline-6-sulfonic acid)
ANOVA: Analysis of variance
BKB: Blackberry
BLB: Blueberry
CAA: Cellular antioxidant activity
DCFH_2_: 2′,7′-dichlorodihydrofluorescein
DCFH-DA: 2′,7′-dichlorofluorescin diacetate
DE: Dried extract
DMEM-advanced: Dulbecco’s modified Eagle’s medium
DMSO: Dimethyl sulfoxide
DPPH: 1,1-Diphenyl-2-picryl-hydrazyl
EC_50_: Median effective concentration
FRAP: Ferric reducing antioxidant power
GA: Gallic acid
GAE: Gallic acid equivalent
HBSS: Hank’s Balanced Salt Solution
IC_50_: Half-maximal inhibitory concentration
MP: *Melicope ptelefolia*
MP-EA: MP ethyl acetate extract
MP-HX: MP hexane extract
MP-MeOH: MP methanol extract
MP-W: MP water extract
MTS: [3-(4,5-dimethylthiazol-2-yl)-5-(3-carboxymethoxyphenyl)-2-(4-sulfophenyl)-2H–tetrazolium]
PBS: Phosphate buffered saline
PP: Polyphenon-60
QN: Quercetin
RB: Raspberry
TFC: Total flavonoid content
tHGA: 2,4,6-trihydroxy-3-geranyl-acetophenone
TPC: Total phenolic content
TPTZ: 2,4,6-tripyridyl-s-triazine
TX: Trolox
z-VAD-fmk: N-Benzyloxycarbonyl-Val-Ala-Asp(O-Me) fluoromethyl ketone
