# Supplementary figures and images for: Melicope ptelefolia leaf extracts exhibit antioxidant activity and exert anti-proliferative effect with apoptosis induction on four different cancer cell lines
Source: BMC Complement Altern Med. 2017 May 5;17:252. doi: 10.1186/s12906-017-1761-9 (PMC5420124; doi:10.1186/s12906-017-1761-9)

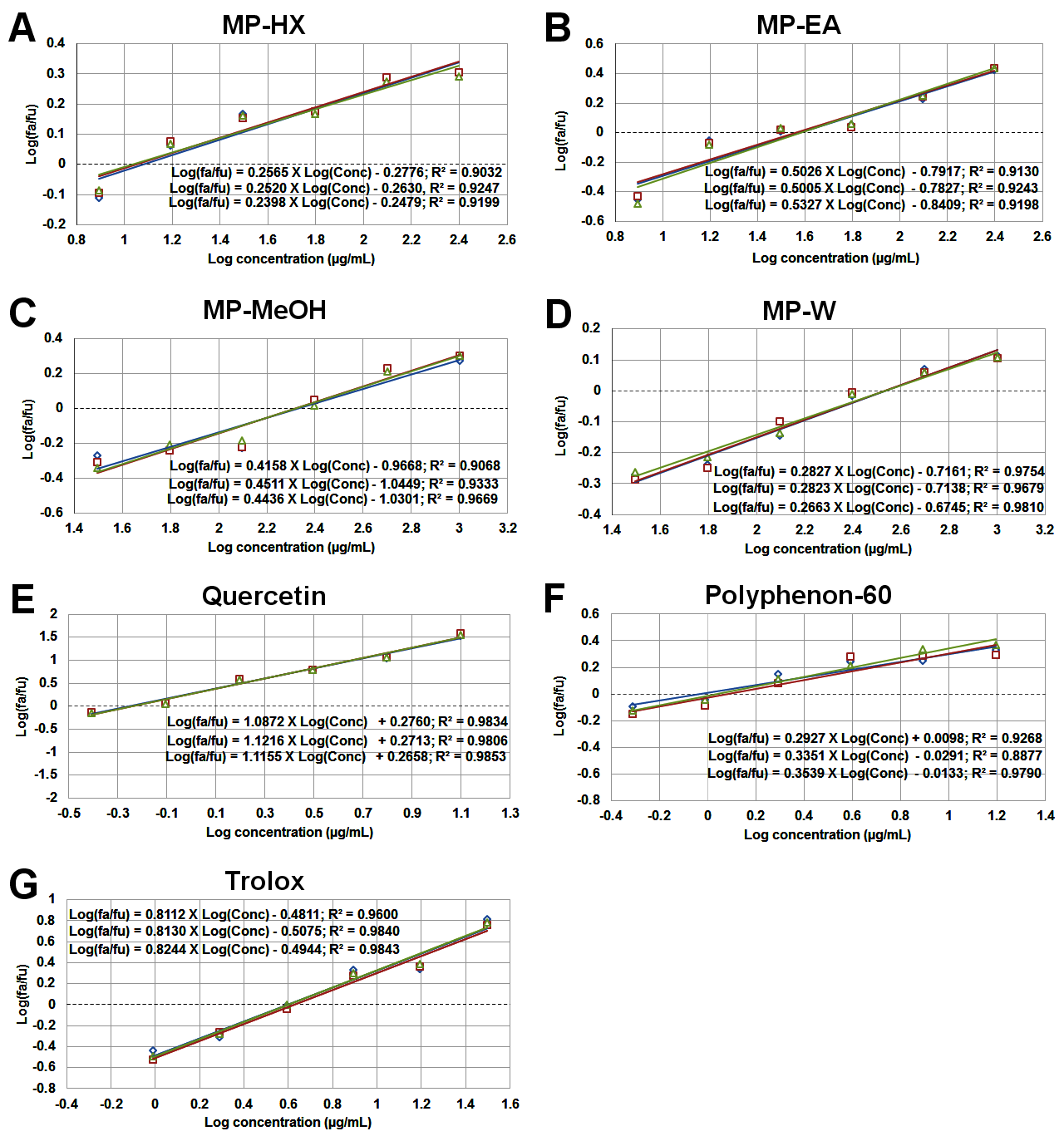

Supplement: Supplementary file 1 — Median effect plots for inhibition of peroxyl radical-induced DCFH2 oxidation in Hs27 cells by (A) MP-HX, (B) MP-EA, (C) MP-MeOH, (D) MP-W, (E) Quercetin, (F) Polyphenon-60 and (G) Trolox. The curves shown in each graph are from a single experiment (mean ± SD, n = 3). (TIFF 228 kb) [file 12906_2017_1761_MOESM1_ESM.tif]

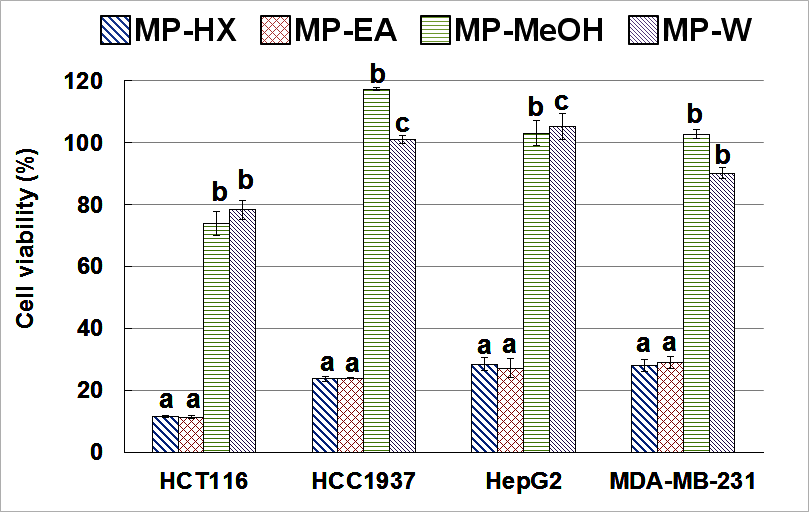

Supplement: Supplementary file 2 — High dose MTS cell viability assay. Effect of MP leaf extracts (250 μg/mL, 48 h) on the cell viability of HCT116, HCC1937 and HepG2 and MDA-MB-231 cell lines. Values are mean ± SD (n = 3). The values with different letters (a-c) are significantly different, p < 0.05. (TIFF 65 kb) [file 12906_2017_1761_MOESM2_ESM.tif]
